# Restoring Discolored Teeth

**Published:** 1889-07

**Authors:** W. A. Barrows

**Affiliations:** Buffalo, N. Y.


ARTICLE IV.

### RESTORING DISCOLORED TEETH.

BY W. A. BARROWS, D. D. S., BUFFALO, N. Y.

There is no subject pertaining to dentistry that receives so little attention at dental meetings, perhaps, as that of bleaching teeth. Presumably, this is due to the fact that the process forms so small a part of daily practice as scarcely to be thought of at professional gatherings where other subjects of greater interest naturally occupy the attention of those present. Again, there are those who are quite skeptical regarding the possibility of restoring badly discolored teeth and to such the topic would have no interest of course,—they know little and care less.

But while it is true that cases requiring such treatment do not often present themselves, yet *when* they do it is a convenience to know *what* to do, or having failed with one method to be acquainted with others.

In addressing you upon this theme it is with no expectation that I shall offer you anything especially new—wish merely to bring to your notice a few agents employed in the bleaching process and the manner in which they are used.

First, it may be proper to refer briefly to the cause of discolored teeth—and right here let me say that the discoloration owing to the use of oxydizable metals in filling is a branch of the subject with which we have nothing to do at this time.

Chiefly then, the primary cause is attributable to what

Harris, Taft and other authors designate as necrosis, but what in professional parlance—though with less elegance, perhaps—is called "dead pulp." When a pulp dies the crown begins to lose its life-like appearance and becomes what is termed "off color." If the tooth is neglected the change of color continues until a dark brownish hue, or even sometimes a bluish cast is reached. This condition is ascribed by some to the presence of disorganized matter in the pulp chamber and its absorption by the surrounding walls of dentine. Another theory, is, that "teeth are darkened by the presence of iron in the cruorin of the blood which when dissolved penetrates the fibrils, thus staining the dentine. Opinions vary, and whichever theory may be the correct one, the discoloration gets there all the same, and the question then presents itself: How shall it be removed? Many agents are employed for this purpose—nitric, sulphurous, oxalic, tartaric, and acetic acids, peroxide of hydrogen and chlorine, but the greatest bleacher of all perhaps, is chlorine; at any rate it is most generally used and proves sufficient in a majority of cases. As to the manner of applying these agents together with the general treatment, a few methods are here described.

No. 1. First apply the rubber dam and cut away as much of the discolored dentine as the strength of the tooth will permit. When the cavity has been otherwise prepared for filling, take a small pledget of cotton dipped in Labarraque's solution, touch to a little powdered alum and insert in the cavity. This should be allowed to remain ten, or fifteen minutes, then removed and the cavity washed out and dried. The application should be repeated as many times as may be necessary to secure the desired result. The alum is used because the chemical action which follows liberates free chlorine that is then nascent and acts more powerfully and rapidly than it would in solution alone.

No. 2. This is the method of Dr. James Truman, of Philadelphia. He is very particular about the form and kind of instruments used, and believes the socket handles with gold, or platinum, bits the best. The latter materials are used to avoid the deleterious action of acids. Instruments of hard wood, or ivory will be found nearly as good with the additional merit of being much less expensive. But it *must be borne in mind that no steel instrument can be brought in contact* with the tooth in this *operation*. The tooth should be thoroughly cleansed of all foreign matter and then the rubber dam adjusted. Dr. Truman uses a ten per cent solution of tartaric acid in connection with chloride of lime. He dips an instrument into the acid and then in the lime and inserts it in the cavity as rapidly as possible. When the cavity is full it should be quickly sealed with gutta-percha and allowed to remain for several days. When the patient returns, the cavity should be thoroughly washed out with distilled water. If the proper color has not been obtained, the process should be gone through with again and repeated until satisfactory results are secured. Chlorine is liberated by all the acids—very rapidly by tartaric, and by sulphuric in less degree.

No. 3. This is Dr. Frank Abbott's method. In reply to my letter of inquiry, he says: " I seldom bleach a tooth, strictly speaking, but depend for a change of color more upon the removal of the discolored dentine and the lining of the crown with some zinc cement which nearly, or quite restore the color. The best bleaching material I have ever tried (chemically speaking) is chloride of lime packed into the cavity, then saturate a pellet of cotton with dilute vinegar (vinegar and water, equal parts) and force it into the centre of the chloride of lime; cover it over with a bit of gutta-percha and wax and let it remain until you see a change in the color of the tooth. This will perhaps be two minutes, possibly longer. Or you may remove it after a few minutes and make another application same as before. Two such applications are as many as a tooth will usually stand without considerable injury. For this reason I generally prefer the method of cutting away the tooth inside and using the white, or light yellow, cement to restore color. The color will with this treatment remain as you fix it, while the dark color often returns after *bleaching*. Use chloride of lime with a wooden spatula. It is better to fill immediately.

No. 4. This is one of the methods of Dr. A. W. Harlan, of Chicago, and which has recently been published in the *Dental Review*.

After the root has been filled and the tooth is free from all tenderness, apply the dam, dry the cavity, and remove all discolored decay. Wash the cavity several times with peroxide of hydrogen and place a few crystals of chloride of alumina in it, moisten them with peroxide, and wait from three to five minutes, then wash the cavity thoroughly with distilled water and apply a solution of thirty grains of borax to the ounce of water until the acid is entirely neutralized. Dry the cavity with hot air and paint the interior with copal-ether varnish. When this is dry, mix oxychloride of zinc of the desired color and fill the cavity full ; allow it to harden then prepare the cavity for the gold filling and fill at once. A cause of failure is the performance of the operation on different days, thereby allowing moisture from without to gain entrance to the cavity and contaminate the oxychloride. Never use a steel instrument when mixing it.

No. 5. Method of Dr. Robert Huey, of Philadelphia. The tooth must be entirely cleared of all decayed matter throughout the length of the canal and pulp chamber. Follow this with a wash of aqua-ammonia, as it has been found that any portion of oily matter interferes with the action of the chlorine. Place the rubber dam on the tooth and fill the upper part of the canal to the foramen. Prepare a solution of oxalic acid—ten grains to the ounce of water. Have ready the very best chlorinated lime—dip instrument in the acid, then in the lime and carry into the tooth as far as possible. Continue until cavity is full. In the course of five minutes remove and refill and so continue for an hour. Usually finish in one sitting. It is necessary to fill temporarily after bleaching and cement is preferred to guttapercha, but the cement should not be inserted until all parts of cavity and canal have been dried by hot air syringe.

No. 6. Dr. W. H. Atkinson's method: Oxalic acid crystals should be forced into the cavity after excavating all that can be spared for strength, then wet with distilled water so as to dissolve the acid in situ, thus affording contact with the salt in the nascent state, converting the dark salts of iron into an oxalate which is white. Whenever staining depends upon vegetable extracts, chlorine is the proper agent to use to bleach. Chloride of lime, Labarraque's solution and chlorine gas are the forms in which to apply it. The gas is the only one that is dangerous and must be cautiously handled, so as not to injure the patient. In all cases of open fistula the filling may be proceeded with at once after arriving at the proper color. My method is to always fill about half of the length of the root from the apex with gutta-percha, or gold, before proceeding to bleach.

In the April number of the *Independent Practitioner*, there was reported an incident of office practice, entitled, "A novel method of bleaching." It seemed, indeed, so entirely novel to me, that I immediately wrote to Dr. Scofield, asking him if that was the method he usually adopted in bleaching teeth. He replied at some length, but in substance stated his belief that it is possible to bleach any tooth in this way provided it has received no previous treatment. Or in other words, he should not expect success when an acid had been already used in attempting to bleach. He thinks the iodine takes up the iron which is in the nerve fibrils and says the tooth must become discolored throughout by the iodine to insure success.

Gentlemen, out of all these different methods, a few "pointers" may be secured that will apply in most cases, perhaps, and contribute in a considerable degree to the success of the operation.

First, adjust the rubber dam and of sufficient size to protect the patient.

Second. Be absolutely thorough in the removal of all decayed and even discolored dentine.

Third. Whatever bleaching agent you may select, bleach the *whole tooth*—root and crown.

Fourth. Having secured the desired color, line the cavity with oxy-chloride of zinc and then fill with gold.

Fifth. While applying the "bleacher," or while mixing the cement, use no steel instruments.

Sixth. *Perform the whole operation at one sitting.*

